# Accurate Behavioral Simulator of All-Digital Time-Domain Smart Temperature Sensors by Using SIMULINK

**DOI:** 10.3390/s16081256

**Published:** 2016-08-08

**Authors:** Chun-Chi Chen, Chao-Lieh Chen, You-Ting Lin

**Affiliations:** Department of Electronic Engineering, National Kaohsiung First University of Science and Technology, Kaohsiung 811, Taiwan; frederic@nkfust.edu.tw (C.-L.C.); u0452804@nkfust.edu.tw (Y.-T.L.)

**Keywords:** CMOS, all digital, time-domain, smart temperature sensor, SIMULINK, HSPICE

## Abstract

This study proposes a new behavioral simulator that uses SIMULINK for all-digital CMOS time-domain smart temperature sensors (TDSTSs) for performing rapid and accurate simulations. Inverter-based TDSTSs offer the benefits of low cost and simple structure for temperature-to-digital conversion and have been developed. Typically, electronic design automation tools, such as HSPICE, are used to simulate TDSTSs for performance evaluations. However, such tools require extremely long simulation time and complex procedures to analyze the results and generate figures. In this paper, we organize simple but accurate equations into a temperature-dependent model (TDM) by which the TDSTSs evaluate temperature behavior. Furthermore, temperature-sensing models of a single CMOS NOT gate were devised using HSPICE simulations. Using the TDM and these temperature-sensing models, a novel simulator in SIMULINK environment was developed to substantially accelerate the simulation and simplify the evaluation procedures. Experiments demonstrated that the simulation results of the proposed simulator have favorable agreement with those obtained from HSPICE simulations, showing that the proposed simulator functions successfully. This is the first behavioral simulator addressing the rapid simulation of TDSTSs.

## 1. Introduction

Computer-aided design (CAD) tools are often used to facilitate electronic circuit design before implementation. They are developed to achieve numerous goals such as the reuse of design components, ease of design modification, automatic generation of designs, and verification of designs against specifications and design rules. The ultimate purpose is to derive predictions of circuit behavior that are highly similar or even identical to the real physical behavior of the circuit after implementation. For analog or mixed-signal integrated circuit (IC) designs, electronic design automation (EDA) tools such as HSPICE are widely used to accurately simulate the ICs for performance evaluations. This transistor-level simulator can derive circuit information precisely, enabling designers to predict the functionality, power dissipation, timing, and reliability of their designs. Although this class of transistor-level simulators is powerful for evaluating circuits for various performance metrics, the simulation process is time-consuming. Generally, this would require at least several hours or possibly several days, depending on the simulation settings. To overcome this problem, at the early design stage of numerous ICs, such as phase locked loops (PLLs), frequency synthesizers, and delta-sigma modulators, behavioral models have been built and evaluated using SIMULINK; some simulation precision was sacrificed for the rapid calculation of rough results [1,2,3,4,5,6,7,8]. Researchers developed so-called “behavioral simulation techniques”, which trade accuracy for speed; the design procedure can thus be accelerated by applying these high-speed simulation techniques. Typically, circuit trimming techniques are further applied during the design of physical device models to mitigate the gap between behavioral and transistor-level simulations. However, the required time for the trimming process is unpredictable.

In numerous industrial, home, and office electronic devices, smart temperature sensors (STSs) are increasingly required to monitor and manage temperatures. Due to the demand for small, low-dissipation devices, CMOS STSs are highly competitive and have strong appeal. Several CMOS time-domain STSs (TDSTSs) have been developed over the past 10 years [9,10,11,12,13,14,15,16,17,18,19,20]. Compared with CMOS voltage-domain STSs, which have highly favorable accuracy for voltage and process variations [21,22,23,24], CMOS TDSTSs possess the advantages of lower cost and lower circuit complexity. Thus, several TDSTSs have been reported [10,11,12,13,14,15,16,17,18,19,20]. HSPICE simulations are precise but extremely time-consuming, particularly because STS simulations must simulate multiple temperature points to evaluate performance. Thus, researchers must develop a time-efficient and accurate simulation tool that can effectively derive valid temperature information regarding TDSTSs. However, to the best of our knowledge, no behavioral simulator for TDSTSs meets the standards of simulators for PLLs, frequency synthesizers, or delta-sigma modulators. Beyond behavioral simulation, accurate predictions are also desirable in transistor-level simulations of TDSTS designs. With such predictions, designers can modify a TDSTS at the early design stage without several rounds of time-consuming HSPICE simulations. 

The widely used SIMULINK platform provides time-efficient behavioral simulations. Thus, this study proposes a high-speed SIMULINK-based behavioral simulator for TDSTSs. In this paper, we organize simple and accurate equations describing TDSTS into a temperature-dependent model (TDM) and derive temperature-sensing models of a CMOS NOT gate by using HSPICE, which has a very short simulation time. With the TDM and the derived models, the proposed simulator achieved rapid and accurate simulation. The remainder of this paper is arranged as follows: Section 2 introduces the TDM of the TDSTSs, and the building blocks of the proposed SIMULINK simulator are introduced in detail in Section 3. Subsequently, Section 4 presents the experimental results, which validate the proposed technique, and finally, Section 5 concludes this study.

## 2. TDM for the TDSTSs

In a TDSTS, an inverter-based delay line or oscillator is typically adopted to sense temperature because a simple CMOS NOT gate can act as an effective proportional to absolute temperature (PTAT) sensor that generates a temperature-dependent delay time *t_NOT_*(*T*) [9,12,13,14,15,16,17], which can be expressed as follows [13,17]:

(1) $$t_{NOT}(T)=\frac{2LC_{L}T_{0}^{km}}{\mu_{0}WC_{ox}V_{DD}}\times \frac{\text{ln}(3-4V_{th}/V_{DD})}{1-V_{th}/V_{DD}}\times \frac{1}{T^{km}}=\gamma \times T^{-km}$$

where *μ*_0_, *T*, *T*_0_, *V_th_*, *W*/*L*, and *C_L_* are the reference carrier mobility, operation temperature, reference temperature, threshold voltage, effective aspect ratio of transistors, and loading capacitance of the NOT gates, respectively. Without considering the effect of voltage variation, the summed parameter *γ* can be regarded as a process-dependent factor that is nearly independent of temperature. The parameter −*km* is considered to be temperature independent; this parameter determines the thermal characteristics of the CMOS NOT gate. For example, its value ranges from −1.2 to −2.0 for a 0.35-µm CMOS process and, thus, the *t_NOT_*(*T*) changes linearly with the temperature [17]. 

**Figure 1:**
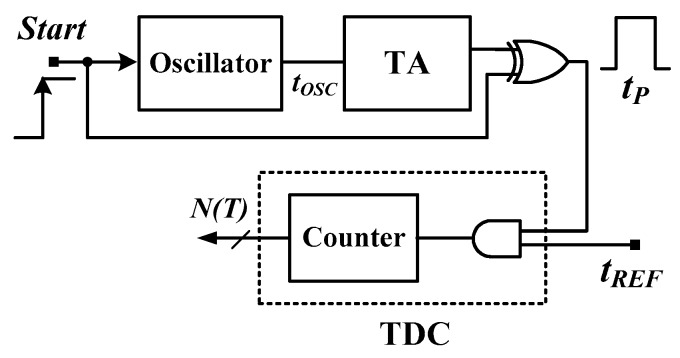
Building block of a simple CMOS TDSTS.

Figure 1 shows a block diagram of an all-digital CMOS oscillator-based TDSTS [12]. An oscillator consists of an odd number (*k*) stages of NOT gates that generate a PTAT period width *t_OSC_*(*T*). To achieve a satisfactory temperature resolution (*R*), a time amplifier (TA) is used to amplify the *t_OSC_*(*T*) with a time gain *n*. With a simple XOR gate, a sufficiently wide PTAT pulse *t_P_*(*T*) is generated. A reference clock with a stable period width (*t_REF_*) is used to convert the *t_P_*(*T*) into a corresponding digital code *N*(*T*) by using an AND gate and output counter. The *N*(*T*) of the simple sensor can be formulated as follows [13,17]:

(2) $$N(T)=\frac{t_{p}(T)}{t_{REF}}=\frac{n\times t_{OSC}(T)}{t_{REF}}=\frac{n}{t_{REF}}\times 2\times k\times \gamma \times T^{-km}$$

Since (in theory) *n*, *k*, and *t_REF_* are ideally temperature- and process-insensitive, the thermal characteristics of *N*(*T*) are ideally identical to those of *t_NOT_*(*T*). Equation (2) is a simple, but accurate, equation that describes the temperature behavior of the TDSTS, which has been verified in previous studies [12,13,17]. If the models (i.e., *γ* × *T*^−*km*^ for process and temperature variation) of a single CMOS NOT gate can be derived precisely, the sensor code *N*(*T*) can be attained by computing Equation (2) such that the performance can be roughly evaluated and the design procedure can be simplified. Various crucial specifications, such as the limits of accuracy and the resolution, can be estimated quickly. 

**Figure 2:**
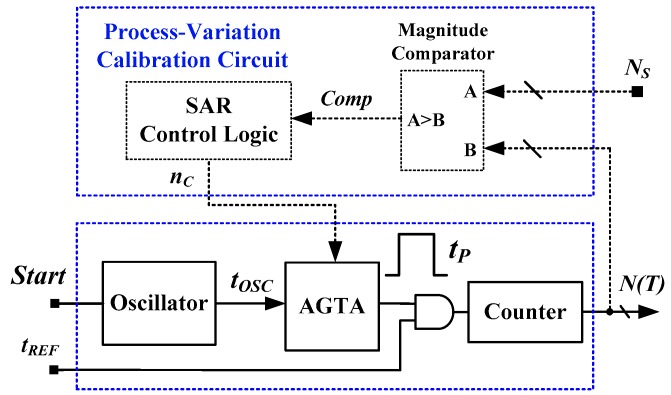
Block diagram of a TDSTS with a process-variation calibration circuit.

Figure 2 shows a block diagram of an all-digital TDSTS with one-point calibration support that incorporates process-variation calibration [13]. In contrast to the simple TDSTS shown in Figure 1, the adjustable-gain time amplifier (AGTA) shown in Figure 2 is modified from a standard time amplifier to realize the variable time gain *n_C_* for calibration. The calibration circuit is composed of a magnitude comparator and SAR (successive approximation register) control logic; this eliminates the influence of process variation (denoted as *i*) to support one-point calibration. The calibration technique and detailed procedure were presented in [13]. For one-point calibration, the calibrated code *N_C,i_*(*T_C_*) at the calibration temperature *T_C_* can be adjusted to match the calibration value *N_S_*. This calibration condition can be expressed as follows:

(3) $$N_{C,i}(T_{C})=\frac{n_{C,i}\times t_{OSC,i}(T_{C})}{t_{REF}}=N_{S}$$

The oscillation period width of the *i*th sensor on *T_C_* (*t_OSC,i_*(*T_C_*)) is compensated dynamically by adjusting the corresponding *n_C,i_*. The calibration result is presented as follows:

(4) $$n_{C,i}=\frac{t_{REF}\times N_{S}}{t_{OSC,i}(T_{C})}=\frac{t_{REF}\times N_{S}}{2\times k\times \gamma \times T_{C}^{-km}}$$

Finally, Equation (4) is substituted into Equation (2), and the *N_C,i_*(*T*) after the calibration can be expressed as:

(5) $$N_{C,i}(T)=\frac{n_{C,i}\times 2\times k\times T^{-km}}{t_{REF}}={\left(\frac{T}{T_{C}}\right)}^{-km}\times N_{S}$$

In other words, with the calibration, the calibrated time gain *n_C,i_* replaces the fixed *n* in the uncalibrated sensor to generate the *N_C,i_*(*T*) of the calibrated sensor. The result of Equation (5) reveals that the process term *γ* can be eliminated effectively, enabling the calibrated sensor to support one-point calibration. According to the two chosen values (*N*(*T*_1_) and *N*(*T*_2_)) and their temperature interval (*T*_1_ − *T*_2_), the sensor resolution *R* can be determined as follows:

(6) $$R=\frac{T_{1}-T_{2}}{N(T_{1})-N(T_{2})}$$

Furthermore, the conversion time can be estimated as the product of the digital value at the highest temperature and *t_REF_* (i.e., *N*(*T*_2_) × *t_REF_*) without considering the delay time of the devices in the sensor and, thus, the ideally lowest conversion rate (*CR*) is determined as:

(7) $$CR=\frac{1}{N(T_{2})\times t_{REF}}$$

The transistor-level of the CMOS TDSTSs can be simulated using HSPICE to derive the uncalibrated codes *N_i_*(*T*) accurately with process and temperature variations (i.e., Equation (2)). With the calibration, the calibrated *n_C,i_* (i.e., Equation (4)) can be simulated to further derive the calibrated codes *N_C,i_*(*T*) (i.e., Equation (5)). The HSPICE simulation is precise but extremely time-consuming. To reduce the simulation time substantially, Equations (1)–(5) were used in the proposed simulator as a TDM for behavioral simulation.

## 3. Proposed Behavioral Simulator Using SIMULINK

According to Equations (1)–(5) of the TDM, the *t_NOT_*(*T*) is the most crucial item because it is the only quantity related to the temperature and process. This feature can be used to develop a time-efficient behavioral simulator for TDSTSs in order to estimate behavior without using HSPICE, significantly reducing the simulation time. Another vital issue is that the simulator is concise because its equations are simple and linear. The equations have been verified to function effectively [12,13,14,15,16,17], which is conducive to precise simulation.

**Figure 3:**
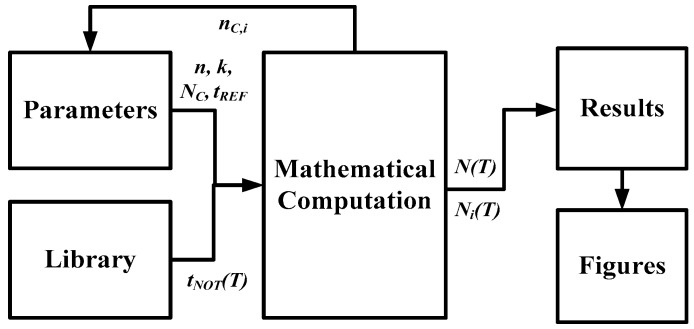
Conceptual block diagram of a concise simulation tool for a TDSTS.

A conceptual block diagram of a concise simulation tool for a TDSTS is shown in Figure 3. The designer selects the basic parameters (*n*, *k*, *t_REF_*, *N_C_*, *T_C_*) and loads the models (*γ* × *T*^−*km*^) from the library for mathematical computation; the system calculates the equations using the models and the input parameters. After the simulation results have been generated, the performance can be evaluated. Moreover, the figures for the results (*N_i_*(*T*) and *N_C,i_*(*T*)) and the processed data (for example, the inaccuracy) can be directly displayed using a suitable program, which simplifies the operations and increases the functionality of the tool.

**Figure 4:**
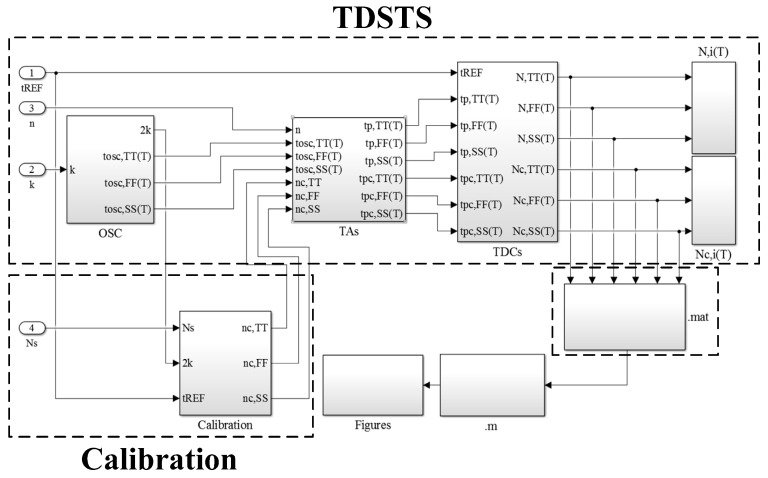
Constructed block of the proposed simulator in the SIMULINK environment.

The objective of this study was to develop a concise and low-cost simulator for performing rapid and accurate TDSTS simulations. SIMULINK is a widely used behavioral simulation platform that is highly suitable for implementing the simulator. Implementation in the SIMULINK environment brings numerous advantages such as high construction flexibility, large computation capability, a user-friendly graphical interface, and extremely short computation time. The design presented in Figure 3 can be elaborated with TDSTSs and process-variation calibration in the SIMULINK environment to yield the constructed block of the proposed simulator shown in Figure 4. The block of the TDSTSs comprises a library that stores the temperature-sensing models of a single NOT gate derived from HSPICE simulations, and two sensors (without and with calibration) for estimating system performance. The block of the calibration is used to generate the calibrated time gain *n_C,i_* for the sensor with calibration. Finally, the simulation results (*N_i_*(*T*) and *N_C,i_*(*T*)) are stored in .mat files for figure generation with MATLAB. The software structure is concise and the defining equations are simple. Thus, a low-cost simulator for rapid performance evaluations can be constructed. 

First, considering the uncalibrated sensor (i.e., the simple sensor without calibration), the input values of the parameters are *k* for determining the oscillation period width *t_OSC,i_*(*T*) (*i* denotes the three process corners: typical-typical (TT), fast-fast (FF), and slow-slow (SS)), *n* for amplifying the width to generate *t_P,i_*(*T*), and *t_REF_* for time-to-digital conversion. The results of *N_i_*(*T*) are obtained using Equation (2) and the models of *t_NOT,i_*(*T*). Second, the process-variation calibration is activated by giving the value of *N_S_* on *T_C_*. Through Equation (3), the corresponding *n*_C,*i*_ of the three process corners can be generated for the AGTA in the calibrated sensor. Then, the estimation of the calibrated sensor is performed. With the same *t_NOT,i_*(*T*), *k*, and *t_REF_*, the computation of Equation (5) generates the results of *N_C,i_*(*T*).

After the results have been generated, the data of *N_i_*(*T*) and *N_C,i_*(*T*) are stored in a .mat file, which is loaded into a prewritten MATLAB file to estimate the sensor resolution *R* by using Equation (6), and to generate the figures that display the performance. In other words, the linearity and inaccuracy after data processing can be directly presented in the figures in a simple manner. This simplifies the figure generation procedure considerably.

### 3.1. Temperature-Sensing Model of a Single CMOS NOT Gate

In the SIMULINK platform, the blocks are described by the equations of the TDM, which express their outputs in terms of the input parameters and NOT gate models. Therefore, the precision of the behavioral simulation depends on how accurately those equations describe the actual condition of each building block and the precision of the captured model. The accurate equations are presented in previous subsections. This subsection introduces the temperature-sensing model of a NOT gate.

To build a library that stores the models for behavioral simulation, an oscillator composed of 21 stages of CMOS NOT gates (PMOS: W/L = 2/2 µm, NMOS: W/L = 1/2 µm) was simulated using HSPICE in a TSMC 0.35-µm CMOS process for process and temperature variations (TT, SS, and FF of the three process corners, ranging from 0 to 80 °C in 10 °C increments). The long MOS channel length was selected to facilitate the realization of a wider oscillation period width. An oscillator with relatively high stages was used to optimize the precision. Furthermore, the models of the single NOT gate were derived by averaging the period width and were stored in the library, as presented in Table 1. This averaging data would be expected to dominate the precision of the proposed simulator. Thus, the numerical precision of the data for the captured models is expected to suffice. It is related to the least significant bit (LSB) time (i.e., *t_REF_*) and the value of 2 × *k* × *n*. For example, Equation (2) shows that when *t_REF_* = 25 ns (i.e., 40 MHz) and 2 × *n* × *k* = 100,000, the precision needs to be in the order of tens of femtoseconds (fs) (i.e., 1/1000 in picoseconds (ps)). This is because when the data are amplified by 100,000, the amplified data of the LSB is near 10 ns at most, which is less than 25 ns (the LSB time). Equivalently, the non-ideal effect for the sensor code is less than 1 LSB and is practically irrelevant to the result. When it is necessary to estimate more results, the data for greater numbers of temperature points in a range wider than the commercial temperature range can be obtained in the same manner.

### 3.2. TDSTSs

**Figure 5:**
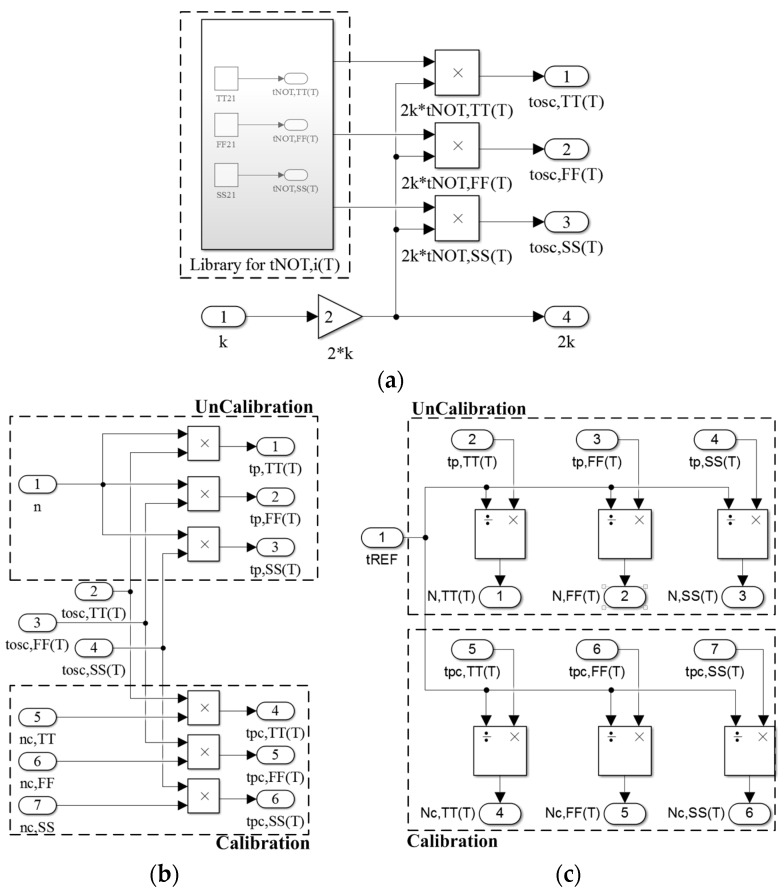
(**a**) Constructed scheme of the oscillator in the SIMULINK environment; (**b**) constructed scheme of the TAs; and (**c**) TDCs in SIMULINK environment.

Figure 5 presents the detailed construction of TDSTSs in the SIMULINK environment for (a) the block of the oscillator including the library; (b) the block of the TAs; and (c) the block of the TDCs. The sensor codes (*N_i_*(*T*) and *N_C,i_*(*T*)) for the two sensors are generated by the two TAs and the corresponding TDCs. Their operation is totally identical except for *n* and *n_C,i_*. Although two sensors were designed in this simulator, the total simulation time was not increased considerably because the proposed simulator is concise and performed by using SIMULINK, which was a notable advantage of the SIMULINK platform.

As shown in Figure 5a, by the product of 2 and *k* and the captured models in the library, the *t_OSC,i_*(*T*) are generated and used in the two sensors. Simultaneously, the value of 2 × *k* is sent to the calibration block (next subsection). Next, when the *t_OSC,i_*(*T*) is amplified with *n* or *n_C,i_* generated from the calibration block, the *t_P,i_*(*T*) in the uncalibrated sensor and the *t_P_*_C,*i*_(*T*) in the calibrated sensor are produced, as presented in Figure 5b. Figure 5c presents the process generating *N_i_*(*T*) or *N_C,i_*(*T*) by counting *t_P,i_*(*T*) or *t_PC,i_*(*T*), respectively, by using the *t_REF_*. The three steps refer to Equations (2) and (5).

### 3.3. Process-Variation Calibration for the Three Process Corners

**Figure 6:**
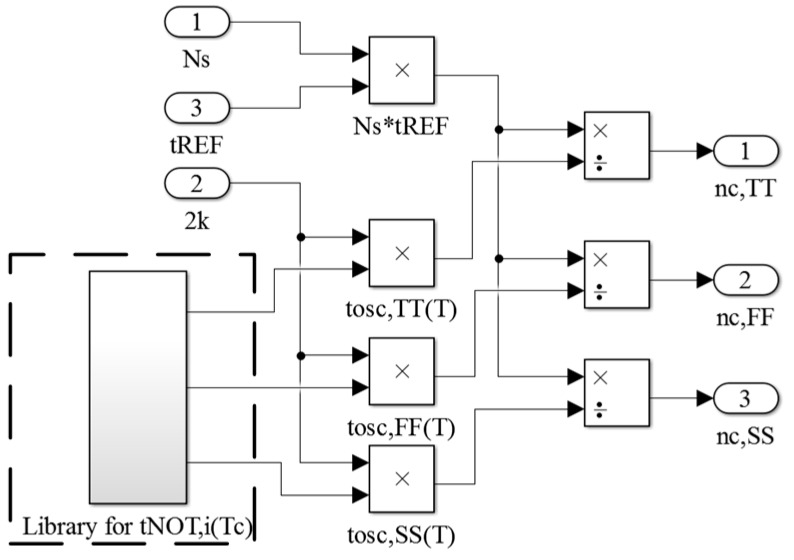
Constructed scheme of the process-variation calibration.

To evaluate the calibrated sensor, the calibrated time gain *n_C,i_* (*n_C,TT_*, *n_C,FF_*, and *n_C,SS_*) must be derived for time amplification. The calibration compensates for the process variation of the oscillator on *T_C_*. Thus, only the model on *T_C_* (i.e., *t_NOT,i_*(*T_C_*)) is used in this scheme. The constructed scheme is illustrated in Figure 6. The value *N_S_* on *T_C_* is selected to perform one-point calibration. The *t_OSC,i_*(*T_C_*) are generated using the value of 2 × *k* (from the block of the oscillator) and the model of *t_NOT,i_*(*T_C_*). With the product of *N_S_* and *t_REF_*, the corresponding *n_C,i_* can be determined using Equation (4) and is used in the calibrated sensor. The higher the value of *N_S_* is, the higher the value of *n_C_* and the sensor resolution are.

### 3.4. Figure Generation for Performance Evaluation

Generally, after a simulation has generated results, a suitable program processes the output into a user-friendly format with figures that present the required functions (e.g., the linearity and the inaccuracy). To simplify the procedure, the simulated data of the proposed simulator are stored in .mat format during simulation and are automatically loaded into a prewritten .m file. MATLAB processes the data in the .m file and generates the required figures. For example, the linearity of the simulation results and the corresponding inaccuracy, including two-point calibration for the uncalibrated sensor and one-point calibration for the calibrated sensor, are often presented in figures to illustrate system performance. Since this simulator features an .m file, the figures of the required functions for the two sensors can be simultaneously exhibited in a remarkably simple manner. Designers can easily see whether the presented performance levels satisfy the specifications.

## 4. Experimental Results

To validate the behavioral simulator, behavioral and transistor-level simulations were performed with the proposed simulator and HSPICE, respectively. A sufficiently wide *t_REF_* can effectively reduce the influence of the delay mismatch and delay variation along various signal paths, thereby improving the precision of the simulation. In addition, a low operational frequency can mitigate high power consumption. Thus, the design should specify relatively wide values of *t_REF_* and *t_OSC_*(*T*). For simplicity, *t_REF_* = 25 ns is given for all situations, and the *T_C_* is set to 40 °C (i.e., the median of 0–80 °C). 

**Figure 7:**
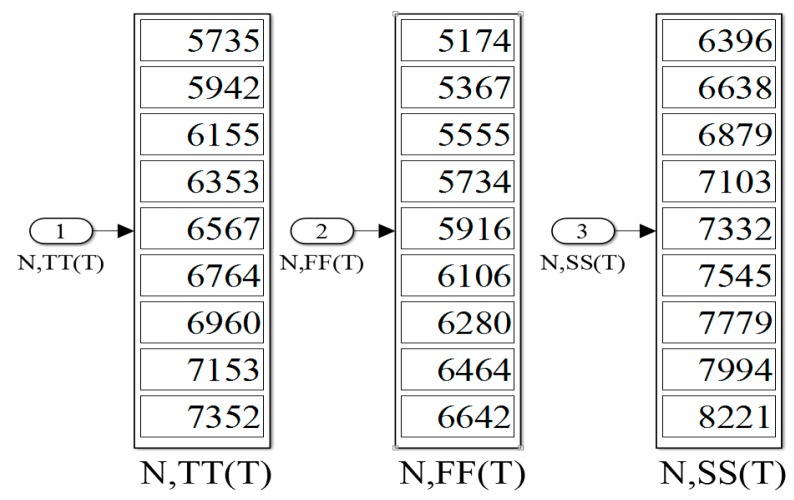
Outcome of *N_i_(T)* in the proposed simulator.

**Figure 8:**
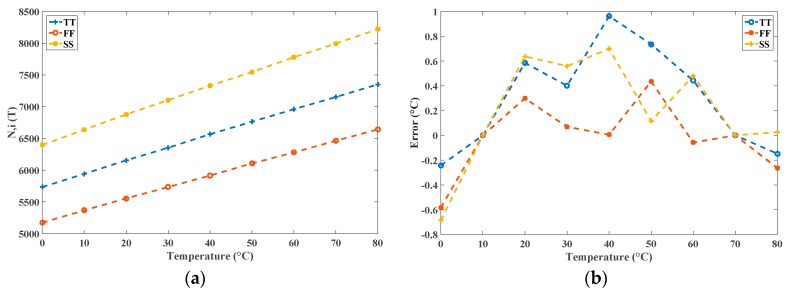
(**a**) Linearity of *N_i_*(*T*) using the proposed simulator; and (**b**) corresponding inaccuracies after two-point calibration of the uncalibrated sensor.

In the uncalibrated sensor, the value *k* = 21 was set to achieve a relatively wide oscillation period width *t_OSC_*(*T*) (for example, 2 × 21 × *t_NOT,TT_* (40 °C) = 32.832 ns) for low power consumption. In additional, the fixed time gain *n* = 5000 was set for a satisfactory *R*. With the transistor-level simulations using HSPICE, the simulation time for the three process corners in 10 °C steps from 0 °C to 80 °C was around 27 hours to derive the results. By the contrast, under the same simulation setting, the total simulation time of the proposed system was only a few seconds, which saved a notable amount of time. After the proposed simulation generated the outcome of *N_i_*(*T*), it was stored in *N_i_*(*T*) .mat; the graphical output is presented in Figure 7. MATLAB loaded the .m file, which contained the data of *N_i_*(*T*), and produced figures depicting the linearity and the inaccuracies after two-point calibration, as shown in Figure 8. Furthermore, the figure generation procedure was simplified considerably. Simultaneously, the proposed simulator calculated the *R* values for the three process corners. For example, in TT mode, *N*(0 °C) = 5735 and *N*(80 °C) = 7352 were chosen with a temperature interval of 80 °C; the *R* was calculated as approximately 0.05 °C. Moreover, the estimated conversion time at 80 °C can be calculated as 183.8 µs (7352 × 25 ns) and the lowest *CR* is determined as 5.44 k samples/s in this TT mode. These operations demonstrated the functionality of the proposed simulator.

**Figure 9:**
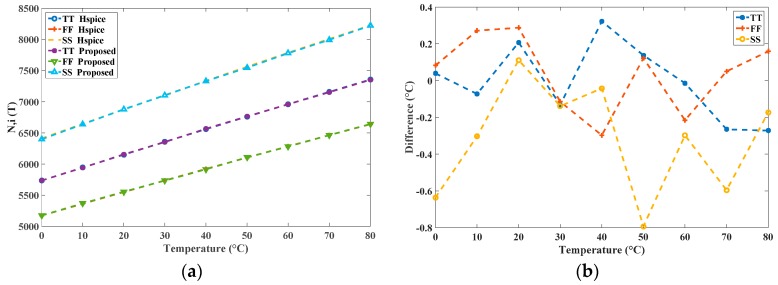
(**a**) Two simulation results *N_i_*(*T*) from the proposed simulator and HSPICE; and (**b**) differences in degrees Celsius.

To further demonstrate the precision of the proposed simulator, the two simulation results *N_i_*(*T*), and their differences in degrees Celsius, are presented in Figure 9 for comparison. The highly similar conditions between the behavioral and transistor-level simulators are shown. The maximal difference is within −0.8 °C~0.35 °C only, which verifies the functionality of the proposed simulator.

**Figure 10:**
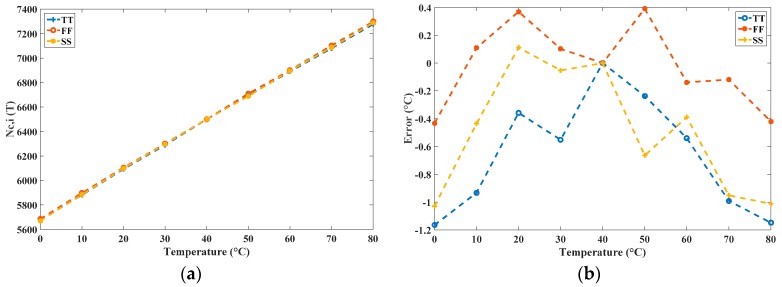
(**a**) *N_C,i_*(*T*) after process-variation calibration by using the proposed simulator; and (**b**) corresponding inaccuracies after one-point calibration for the calibrated sensor.

To perform the process-variation calibration, the value of *N_S_* = 6500 was set to *T_C_* = 40 °C to realize a calibrated *R* of approximately 0.05 °C. The same values of *k* = 21 and *t_REF_* = 25 ns were selected and the model *t_NOT,i_*(40 °C) was used to determine the *n_C,i_* for the three process corners. With the same operation as that of the uncalibrated sensor, the calibrated sensor attained the calibrated *N_C,i_*(*T*) in an extremely short time. The generated figures for the simulated *N_C,i_*(*T*) after the calibration and for the corresponding inaccuracy after one-point calibration are shown in Figure 10. The *N_C,i_*(*T*) values for the three corners nearly coincided and a stable *R* was achieved, thereby validating the proposed one-point calibration.

**Figure 11:**
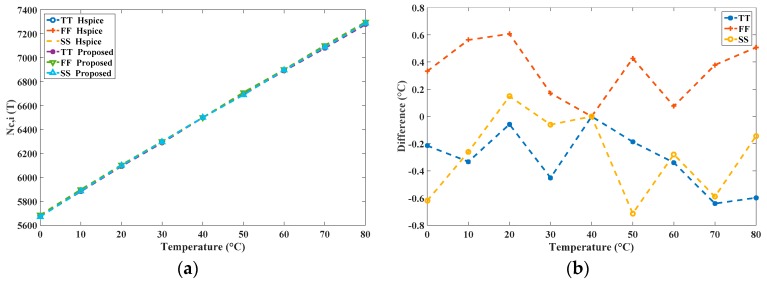
(**a**) Simulation results of the two simulators after calibration; and (**b**) differences in degrees Celsius.

**Figure 12:**
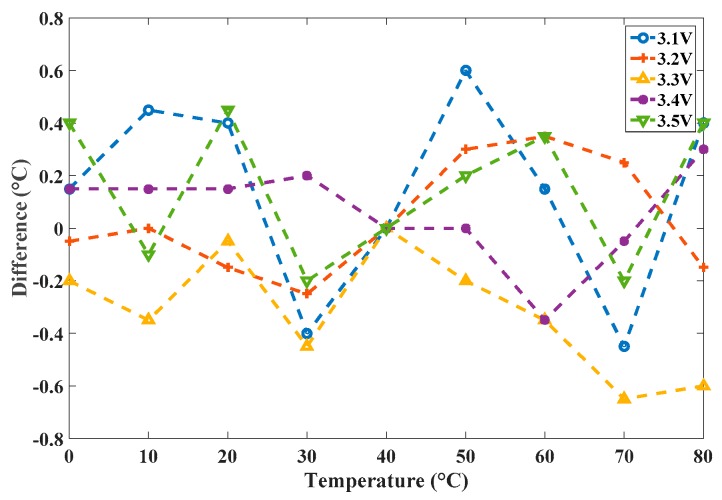
Differences in degrees Celsius for voltage variation.

To verify the precision, a calibration was executed using HSPICE for comparison; this required a substantial amount of simulation time. The HSPICE-simulated *n*_C,*i*_ were used as the corresponding time gain values to simulate a transistor-level calibrated sensor, and (predictably) the simulation time was very long. The simulation results *N_C,i_*(*T*) of the two simulators are shown in Figure 11a and the maximal difference is in the range of −0.7 °C to 0.6 °C, as shown in Figure 11b. The highly similar results of these tests validate the calibration of the proposed simulator. To further verify the effect of the voltage variation, we capture the models of the CMOS inverter from 3.1 V to 3.5 V and store them in the library. With the same operation, Figure 12 shows the corresponding differences and presents the similar results, which validate the proposed simulator for the different-voltage operation.

**Figure 13:**
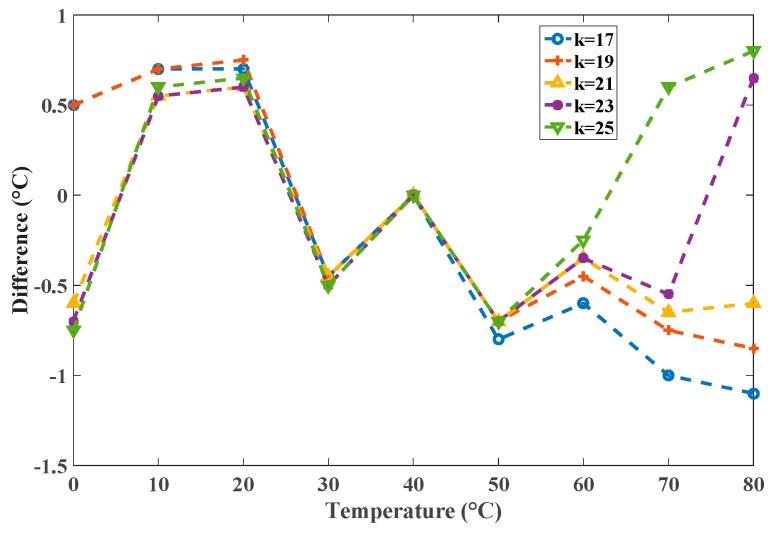
The differences for *k* = 17–25 with the same model.

For precision considerations, the selected value of *k* also determines the precision of the simulator because of the precision of the captured models. Using the models listed in Table 1, simulations of the calibrated sensor were performed using the proposed system and HSPICE with values of *k* that varied from 17 to 25 with increments of 2. The corresponding differences in degrees Celsius are presented in Figure 13. The smallest difference was at *k* = 21; both *k* = 17 and 25 had larger differences than *k* = 21 did. The greater the distance between *k* and 21 was, the greater the difference was. This shows that the captured models for identical stages should be used to achieve the highest possible precision.

**Figure 14:**
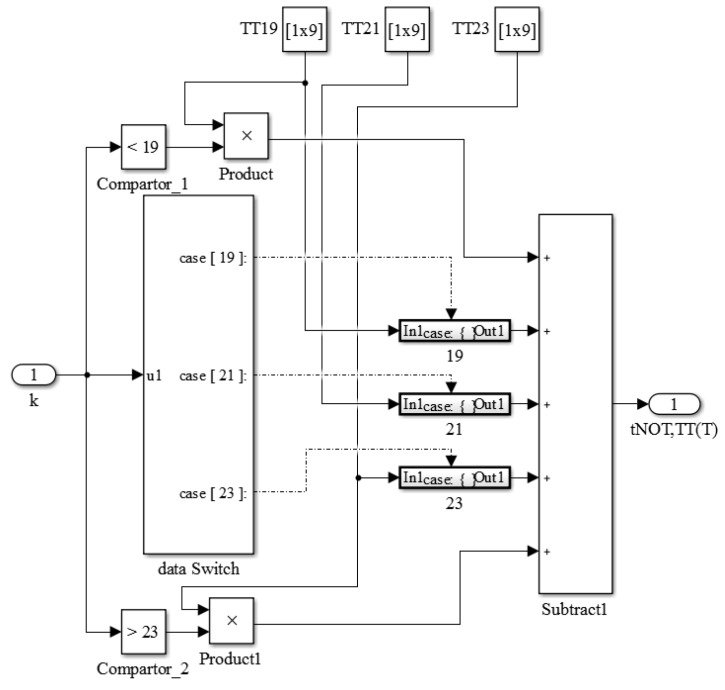
Construction scheme of the modified library with three models.

**Figure 15:**
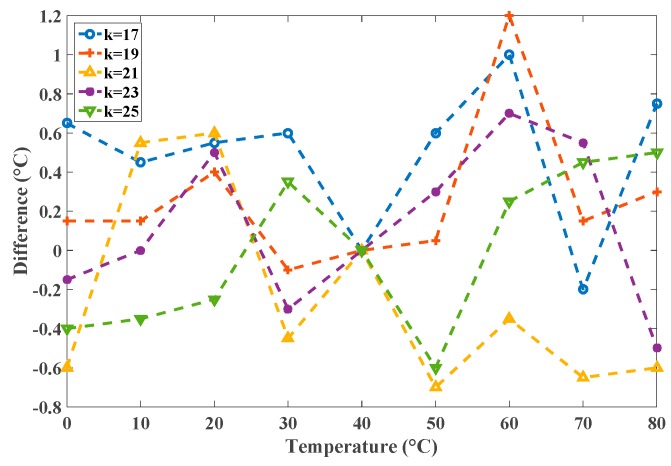
The differences for *k* = 17–25 with the corresponding model.

To improve the precision, the library was extended with models of the appropriate stages (*k* = 17–25). For simplicity, Figure 14 shows the extended library with three TT corner models. In addition to the extended models, the control logics for selecting the *k* values and the corresponding switches were added to output the appropriate model for the oscillator. Additionally, the designer could input *k* > 25 or *k* < 17 and the system would automatically substitute the model for *k* = 25 or *k* = 17, respectively. The improvements from this modification are presented in Figure 15. The precision values were obviously enhanced by this modified library. When necessary, the detailed models for every value of *k* can be constructed to achieve high precision by increasing the library cost. The experiments show that the proposed simulator for all-digital CMOS TDSTSs can perform rapid simulations with accurate results.

## 5. Conclusions

An accurate behavioral simulator of an all-digital CMOS TDSTS based on SIMULINK is presented in this paper. A behavioral simulation tool was developed as a rapid performance evaluator that provides the benefits of a user-friendly graphical interface, high flexibility for circuit extension, and substantial data processing capabilities. With the TDM and the captured models of the unit device, the proposed simulator derives accurate results rapidly. The high precision of the simulator was verified through a transistor-level simulation by using HSPICE. To our knowledge, this is the first behavioral simulator for TDSTSs.

## Figures and Tables

**Table 1:** Simulation model (data) with numerical fineness of tens in digit in fs.

	Process Corner	TT (ps)	FF (ps)	SS (ps)
Temperature				
0 °C		682.71	615.89	761.48
10 °C		707.32	638.92	790.24
20 °C		732.76	661.34	818.87
30 °C		756.34	682.60	845.57
40 °C		781.72	704.23	872.86
50 °C		805.21	726.93	898.20
60 °C		828.53	747.62	926.09
70 °C		851.50	769.51	951.71
80 °C		875.17	790.70	978.69
